# KatharoSeq Enables High-Throughput Microbiome Analysis from Low-Biomass Samples

**DOI:** 10.1128/mSystems.00218-17

**Published:** 2018-03-13

**Authors:** Jeremiah J. Minich, Qiyun Zhu, Stefan Janssen, Ryan Hendrickson, Amnon Amir, Russ Vetter, John Hyde, Megan M. Doty, Kristina Stillwell, James Benardini, Jae H. Kim, Eric E. Allen, Kasthuri Venkateswaran, Rob Knight

**Affiliations:** a Marine Biology Research Division, Scripps Institution of Oceanography, La Jolla, California, USA; b Department of Pediatrics, University of California San Diego, La Jolla, California, USA; c Jet Propulsion Laboratory, California Institute of Technology, Pasadena, California, USA; d NOAA Southwest Fisheries Science Center, La Jolla, California, USA; e Division of Neonatology, University of California San Diego, La Jolla, California, USA; f Center for Microbiome Innovation—Jacobs School of Engineering, University of California San Diego, La Jolla, California, USA; g Department of Computer Science and Engineering, University of California San Diego, La Jolla, California, USA; University of Hawaii at Manoa

**Keywords:** 16S rRNA amplicon, *Acinetobacter*, *Staphylococcus*, *Vibrio*, abalone, built environment, low biomass, metagenomics, microbial ecology, neonatal intensive care unit, NICU

## Abstract

Microbiome analyses of low-biomass samples are challenging because of contamination and inefficiencies, leading many investigators to employ low-throughput methods with minimal controls. We developed a new automated protocol, KatharoSeq (from the Greek katharos [clean]), that outperforms single-tube extractions while processing at least five times as fast. KatharoSeq incorporates positive and negative controls to reveal the whole bacterial community from inputs of as few as 50 cells and correctly identifies 90.6% (standard error, 0.013%) of the reads from 500 cells. To demonstrate the broad utility of KatharoSeq, we performed 16S rRNA amplicon and shotgun metagenome analyses of the Jet Propulsion Laboratory spacecraft assembly facility (SAF; *n* = 192, 96), 52 rooms of a neonatal intensive care unit (NICU; *n* = 388, 337), and an endangered-abalone-rearing facility (*n* = 192, 123), obtaining spatially resolved, unique microbiomes reproducible across hundreds of samples. The SAF, our primary focus, contains 32 sOTUs (sub-OTUs, defined as exact sequence matches) and their inferred variants identified by the deblur algorithm, with four (*Acinetobacter lwoffii*, *Paracoccus marcusii*, *Mycobacterium* sp., and *Novosphingobium*) being present in >75% of the samples. According to microbial spatial topography, the most abundant cleanroom contaminant, *A. lwoffii*, is related to human foot traffic exposure. In the NICU, we have been able to discriminate environmental exposure related to patient infectious disease, and in the abalone facility, we show that microbial communities reflect the marine environment rather than human input. Consequently, we demonstrate the feasibility and utility of large-scale, low-biomass metagenomic analyses using the KatharoSeq protocol.

**IMPORTANCE** Various indoor, outdoor, and host-associated environments contain small quantities of microbial biomass and represent a niche that is often understudied because of technical constraints. Many studies that attempt to evaluate these low-biomass microbiome samples are riddled with erroneous results that are typically false positive signals obtained during the sampling process. We have investigated various low-biomass kits and methods to determine the limit of detection of these pipelines. Here we present KatharoSeq, a high-throughput protocol combining laboratory and bioinformatic methods that can differentiate a true positive signal in samples with as few as 50 to 500 cells. We demonstrate the application of this method in three unique low-biomass environments, including a SAF, a hospital NICU, and an abalone-rearing facility.

## Abstract (precis)

Various indoor, outdoor, and host-associated environments contain small quantities of microbial biomass and represent a niche that is often understudied because of technical constraints. Many studies that attempt to evaluate these low-biomass microbiome samples are riddled with erroneous results that are typically false positive signals obtained during the sampling process. We have investigated various low-biomass kits and methods to determine the limit of detection of these pipelines. Here we present KatharoSeq, a high-throughput protocol combining laboratory and bioinformatic methods that can differentiate a true positive signal in samples with as few as 50 to 500 cells. We demonstrate the application of this method in three unique low-biomass environments, including a SAF, a hospital NICU, and an abalone-rearing facility.

## INTRODUCTION

Many emerging applications of the microbiome, ranging from forensics (1) to medicine to optimization of the cleanliness of facilities where sensitive components are assembled (2, 3), require analysis of low-biomass samples. These applications are moving rapidly away from microbial load measurement and toward the characterization of microbial community composition. Recent studies of the built environment have identified key factors driving the microbiome on surfaces and in air, including the transfer of microbes from humans to environmental surfaces (4), due to the fact that humans can shed 10 million bacteria/h in indoor settings (5). Other key factors modifying the indoor microbiome include temperature, humidity, airflow rates (6), ventilation type (4), and patients or health care workers (7). However, built environments low in microbial biomass remain difficult to study because of poor DNA extraction and amplification efficiency.

Spacecraft assembly facilities (SAFs) are extremely low-microbial-biomass environments, even compared to other built or low-biomass environments such as hospitals (6), pharmaceutical production facilities (8), and indoor environments (3), because the National Aeronautics and Space Administration (NASA) takes extreme steps to avoid the transfer of any terrestrial contaminants to other planets (9). More than 15 years of microbiological surveys of various NASA and European Space Agency cleanroom facilities showed that 1 to 10 m^2^ needs to be sampled to obtain reproducible microbiome signatures by Sanger sequencing, PhyloChip, 454 pyrosequencing, or Illumina sequencing (2). These procedures are critical because NASA allows only swabs, not larger sampling devices, for the collection of materials from sensitive spacecraft hardware surfaces (e.g., components of a sampling or life detection system, typically with surface areas of <1 m^2^). However, many other low-microbial-biomass environments are of considerable interest, including neonatal intensive care unit (NICU) (10, 11) and aquaculture (12) settings, motivating the development of a general technique that works across these environments.

We developed a new protocol, KatharoSeq (from the Greek katharos, meaning clean or pure), that combines high sensitivity and low contamination to study the nature and distribution of the few microbes that survive in low-microbial-biomass settings. Because many popular DNA extraction and amplification kits are contaminated with trace levels of microbial DNA, we reasoned that spiked positive controls would be essential in determining whether amplicon products from low-biomass samples are true reflections of the environment (13, 14). Many investigators have also expressed concern that high-throughput methods introduce well-to-well contamination or reduced efficiency, and thus, laborious and time-consuming single-tube extractions must be performed. KatharoSeq consists of a commercial off-the-shelf high-throughput DNA extraction protocol, combined with carefully arranged titrations of positive and negative controls at the DNA extraction and library construction phase to assess cell counts and well-to-well contamination, together with an integrated bioinformatics pipeline for calculating and applying sample exclusion that is compatible with either amplicon sequencing or shotgun metagenomics.

To demonstrate KatharoSeq’s utility, we applied it to three disparate built environments with low microbial biomass. These samples consisted of 100 25-cm^2^ floor samples and 92 controls from the Jet Propulsion Laboratory (JPL) SAF, 335 locations (primarily 25 cm^2^) across all 52 rooms of the NICU with 53 controls, and 159 sampling points (primarily 25 cm^2^) within an endangered-abalone-rearing facility with 33 controls.

## RESULTS

### Choosing a DNA extraction technique for KatharoSeq.

**FIG 1:**
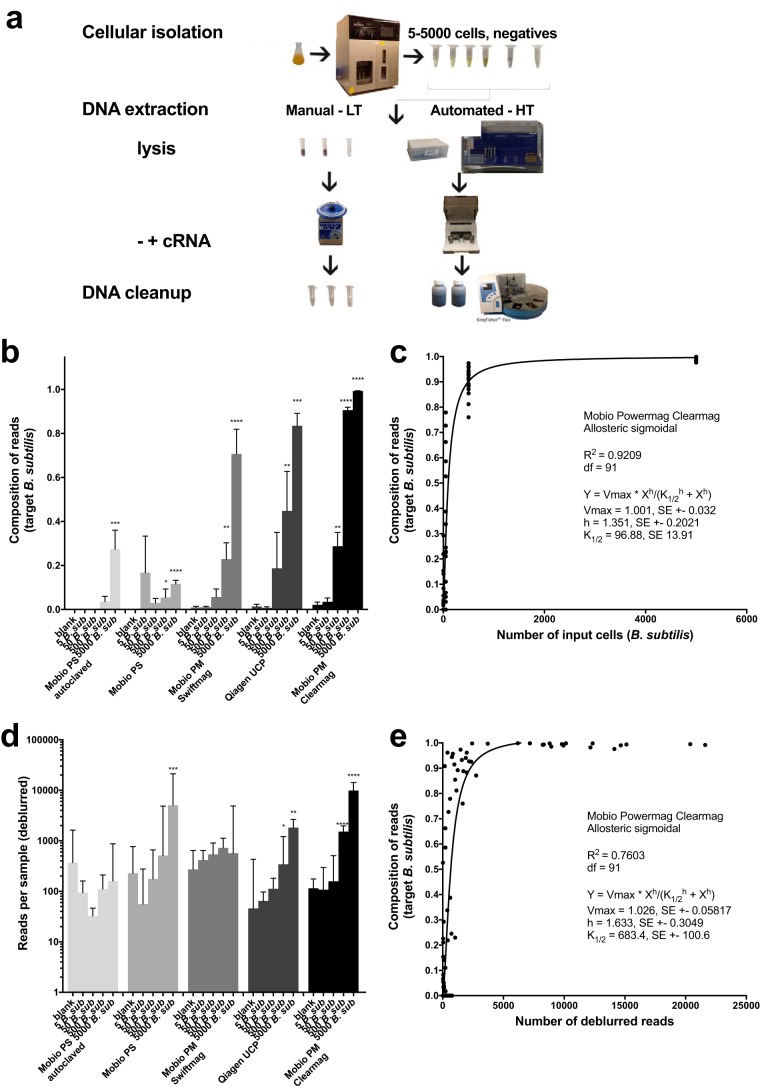
Low-biomass microbiome kit evaluation. (a) Experimental design of low-biomass kit evaluating negative and positive controls (5, 50, 500, and 5,000 bacterial cells) across three solid-phase and two magnetic-bead-based DNA extraction methods. LT, low throughput; HT, high throughput. (b) Libraries of 16S rRNA amplicons were sequenced, and the limit of detection of *B. subtilis* from DNA extraction was determined by comparing the composition of the expected target in known inputs of cells with that in negative controls by a nonparametric Kruskal-Wallis test (Benjamini-Hochberg FDR of 0.05). (c) The limit of detection of the assay and approximate background noise were determined by calculating the *K*_1/2_ value by using the allosteric sigmoidal equation on the *Bacillus* composition. (d) The median read counts (interquartile range) were compared against blanks by using a nonparametric Kruskal-Wallis test (Benjamini-Hochberg FDR of 0.05) to distinguish signal from noise. (e) Read counts were plotted against the expected composition of the target and fitted with an allosteric sigmoidal equation to describe the number of reads where 50% of the read composition was the expected target. This was performed for DNA extraction positive controls from the Mo Bio PowerMag kit, which performed the best, with a limit of detection of 50 cells.

**FIG 2:**
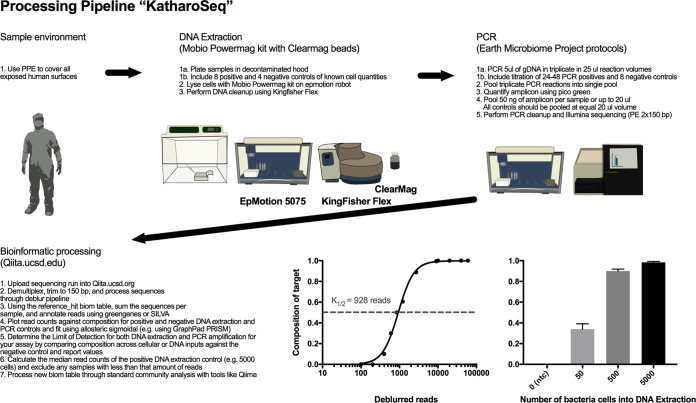
Description of the low-biomass microbiome bench protocol and computational analysis. KatharoSeq-specific recommendations are highlighted throughout the pipeline from sample collection to bioinformatic sample exclusion.

We benchmarked several different high-throughput commercial DNA extraction and bead cleanup techniques and compared them to single-tube extractions by processing negative and positive controls. For positive controls, a single bacterial isolate, *Bacillus subtilis*, was grown and then sorted with a fluorescence-activated cell sorter (FACS) so that a titration of cells (*n* = 5, 50, 500, or 5,000) could be processed and compared across techniques (Fig. 1a). The Mo Bio PowerMag with a ClearMag bead cleanup step consistently performed best, as it had the lowest limit of detection and thus was chosen for KatharoSeq (Fig. 1b). With a 50-cell input, we could differentiate the positive control, *B. subtilis*, from the negative controls, although only 28.8% of the sequences aligned with the target. With a 500-cell input, 90.6% of the reads were mapped to *Bacillus* with all 18 replicates generating sufficient sequences for analysis, thus placing a conservative limit of detection at 500 cells (Fig. 1b). On the basis of these titrations from the Mo Bio PowerMag-with-ClearMag protocol, we determined the total background noise of the entire pipeline (Fig. 2) to be approximately 96.88 cells per sample (*K*_1/2)_ from an allosteric sigmoidal distribution, *R*^2^ = 0.9209 (Fig. 1c). Thus, if a given sample has an initial starting quantity of 96.9 cells, approximately 50% of the reads will be the primary sample while the other 50% will be contaminant. Of the low-throughput methods, the Qiagen Ultraclean Pathogen kit performed best (Fig. 1b).

### Deriving sample exclusion criteria.

Since one of the challenges in low-biomass microbiome analyses is determining how to exclude samples, we also evaluated the read counts across controls from this comparison. The number of reads produced per sample was also highly correlated with the number of input cells (Fig. 1d) and can thus be used as a guide for sample exclusion. When comparing the read counts of positive and negative controls, the Mo Bio PowerMag-with-ClearMag protocol was able to distinguish down to 500 cells, which was the lowest number among the kits tested (Fig. 1d). Upon applying the allosteric sigmoidal equation to the Mo Bio PowerMag-ClearMag samples, we determine that at *K*_1/2_, when 50% of the reads from the positive control mapped to the target organism, the read count was 683 (Fig. 1e). Thus, in the low-biomass experiment, one could exclude samples with <683 reads. All 24 DNA extraction negative controls had <683 reads, while only 1 sample from the 5-cell input and 2 samples from the 50-cell input generated at least 683 reads. Of the 500-cell inputs, 16 of the 18 samples had at least 683 reads and thus would be included in the analysis, while all 18 samples from the 5,000-cell input produced sufficient reads for inclusion. Thus, by using read counts from positive controls paired with the composition of those positive controls, one can determine a per-study limit of detection.

Additional methods attempted to improve the extraction efficiency or quality control (QC) were futile. More specifically, it is common in molecular biology to add carrier RNA during DNA extraction to improve the extraction efficiency or yield, particularly with columns. In this experiment, adding carrier RNA did not affect recovery and detection by the high-throughput methods and only partially increased detection by the low-throughput method (see Fig. S1 in the supplemental material). Gel electrophoresis is a low-cost quality assurance/QC method typically used to test whether enough library product was produced by a PCR or library construction for next-generation sequencing, but the effectiveness of this approach has, to our knowledge, not been assessed for modern library construction protocols and specifically for low-biomass samples. Amplicon libraries from only 26 of the 72 DNA extraction positive controls (5-, 50-, 500-, and 5,000-cell inputs) generated a visible band in a 2% agarose gel, while 37 libraries were shown to have enough reads to be included in the analysis by the KatharoSeq exclusion method (Fig. S2b). This demonstrates that library success based on the presence of a band in a gel has a 30% false-negativity rate and thus should be absolutely avoided for low-biomass applications. Of the 37 successful libraries, 1 was from a 5-cell input, 2 were from 50-cell inputs, 16 were from 500-cell inputs, and 18 out of 18 were from 5,000-cell inputs. None of the DNA extraction negative controls showed a band in the gel, and each of these negative controls generated fewer than 683 reads. From these results, we deem it unwise to utilize gels to QC low-biomass libraries and instead insist on the use of read counts from positive and negative controls.

10.1128/mSystems.00218-17.1 FIG S1  Effects of cRNA addition on the total number of reads (a to e) and limit of *B. subtilis* detection (f to j) across all five kits evaluated. Download FIG S1, TIF file, 0.8 MB. Copyright © 2018 Minich et al. 2018 Minich et al. This content is distributed under the terms of the Creative Commons Attribution 4.0 International license.

10.1128/mSystems.00218-17.2 FIG S2  Low-biomass amplicon library QC. Download FIG S2, TIF file, 0.3 MB. Copyright © 2018 Minich et al. 2018 Minich et al. This content is distributed under the terms of the Creative Commons Attribution 4.0 International license.

We applied this method of exclusion to samples from the three unique cleanrooms. For the subsequent built-environment analyses, we conservatively designated our sample exclusion to be the median read count of the 5,000-cell input. This was determined on the basis of positive controls for each built environment (SAF, 1,696 reads; NICU and abalone-rearing facility, 2,015 reads). Values will vary from experiment to experiment primarily because of differences in overall sequencing depth and, marginally, well-to-well contamination occurring during sample collection or processing and thus should be calculated for each experiment and sequencing run.

### Applying KatharoSeq to low-biomass environments.

We applied KatharoSeq amplicon sequencing to three unique low-biomass environments: the JPL SAF, an NICU, and a facility for rearing critically endangered abalone. Together with positive controls from the initial low-biomass method development, a total of 1,072 samples generated 25,969,842 reads representing 16,417 suboperational taxonomic units (sOTUs). We also processed, 556 samples from the three environments for shotgun metagenomics (96 from the SAF, 337 from the NICU, and 123 from the abalone-rearing facility). The success rate of samples processed through the 16S rRNA amplicon pipeline (SAF, 57%; NICU, 50%; abalone-rearing facility, 90%) was generally higher than the success rate of the shotgun metagenome analysis (SAF, 64%; NICU, 44%; abalone-rearing facility, 35%).

Results from the SAF cleanroom-specific microbiome highlighted the importance of using controls. For 16S rRNA analysis, the minimum exclusion of 1,696 reads was determined by calculating the median read counts of the positive DNA extraction controls at 5,000 cells (Fig. 1a and b). Forty-three samples yielded <1,696 reads and were omitted from the analysis (Fig. 1b).

**FIG 3:**
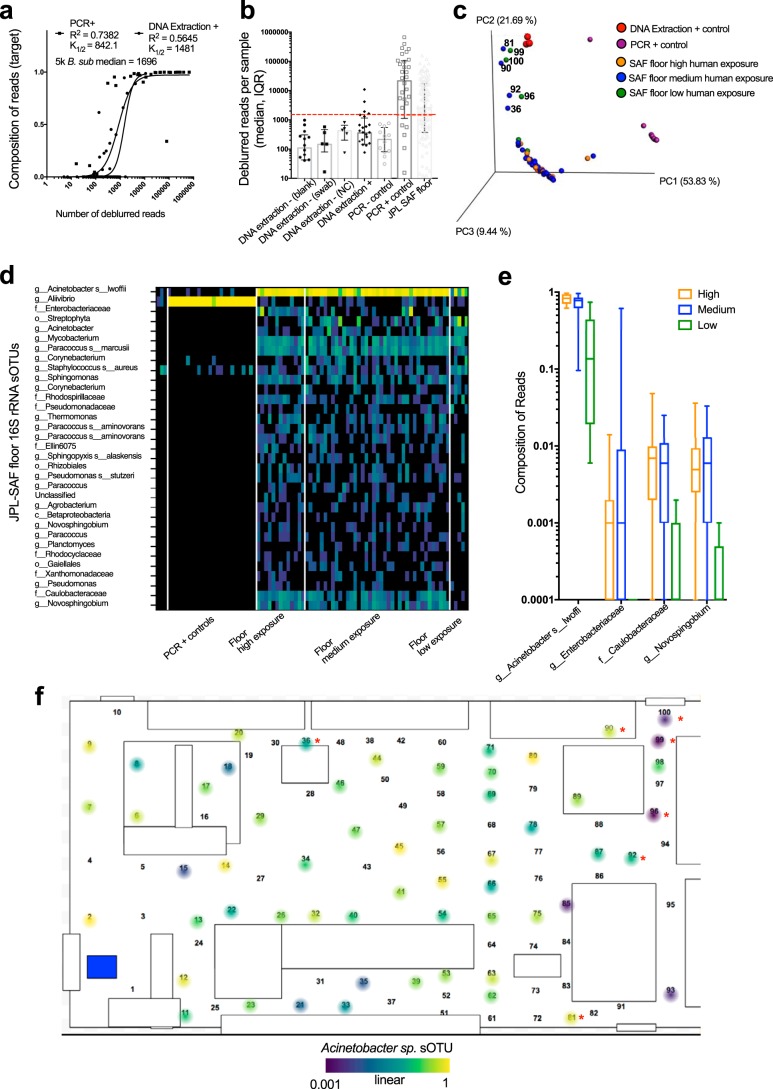
Optimized KatharoSeq protocol applied to a JPL cleanroom facility. (a) The number of deblurred reads represented by the DNA extraction and PCR positive controls is plotted against the composition of the target organism. The distribution is fitted with an allosteric sigmoidal equation that describes the number of reads when 50% of the composition is recorded (*K*_1/2_). (b) The numbers of reads (median and interquartile range) per control type and floor samples are depicted, with the red dotted line indicating the sample exclusion value of 1,696 reads. NC, negative control. (c) Beta diversity PCoA plots calculated from weighted UniFrac distances from the JPL samples (*n* = 59) and controls (*n* = 15) with at least 1,696 deblurred reads, colored by foot traffic frequency (human exposure) and control type. (d) All 32 sOTUs found to be associated with the JPL floor community are shown in a heatmap in comparison with controls. (e) Associations of human foot traffic with sample composition of the top four most abundant sOTUs. (f) The most abundant sOTU in the samples, *A. lwoffii*, is mapped onto the JPL facility 2D map, with the samples deviating from the cluster noted with a red asterisk.

KatharoSeq revealed a SAF cleanroom-specific microbiome with a distinct and interpretable spatial pattern (Fig. 3). Beta diversity analysis revealed that a subset of seven samples clustered in a way that did not match the overall pattern (Fig. 3c), indicating a different microbial composition related to human exposure. When cross-referencing to the facility floor (Fig. 3f, red asterisks), these primarily low-traffic and normal-traffic samples localized to the top right corner and top middle of the facility, a nonrandom pattern distinguishing them from reagent contaminants or sequencing failures.

Four sOTUs were identified by 16S rRNA gene amplicon analysis as frequent members of the JPL SAF microbiome, present in at least 75% of the JPL floor samples. These were *Paracoccus marcusii*, *Mycobacterium* sp., *Novosphingobium*, and *Acinetobacter lwoffii*. *Sphingomonas* sp. was also present in 70% of the JPL floor samples. This organism was identified as a common SAF core sOTU in the Kennedy Space Center (KSC), Johnson Space Center, and JPL (15), increasing confidence in its prevalence in this type of environment.

Thirty-two sOTUs were differentially abundant in the global SAF floor samples, compared to the one sOTU associated with the PCR positive controls (Fig. 3d). Of these 32 SAF-associated sOTUs, 4 were positively associated with high human exposure, as determined by differential abundance, and included *A. lwoffii*, *Enterobacteriaceae*, *Caulobacteraceae*, and *Novosphingobium* sp. (Fig. 3e). The *A. lwoffii* sOTU was present in the negative JPL and positive PCR controls, but the levels were orders of magnitude lower than the real signal, indicating that this result stems from a minimal amount of well-to-well contamination (Fig. 3d). The composition of *A. lwoffii*, when mapped onto the two-dimensional (2D) layout of the JPL SAF, is in lower relative abundance in the top right corner and top middle. This spatial pattern strikingly resembles the overall spatial relationship in the community level beta diversity analysis (Fig. 3c).

Shotgun metagenomic analysis of very-low-biomass samples is very challenging, and most of the research on such samples to date has used amplicon-based techniques or whole-genome amplified products (13). Our floor samples had very low DNA yields, with a mean double-stranded DNA yield of 59.4 (standard error, 9.1) pg/µl, and 23% of the samples had DNA concentrations below the detection limit of 1 pg/µl. We performed metagenomic sequencing of 96 of the 192 samples, including 50 representative JPL SAF floor samples (based on the amplicon analysis) and 46 controls, without the use of whole-genome amplification.

Analysis of the spiked controls provided confidence in the assemblies of other microbes from the samples for both 16S rRNA gene analysis and shotgun metagenomics. As expected, for the *Vibrio fischeri* genomic DNA (gDNA)-spiked controls, there were positive correlations among the starting genome quantity, the number of resulting sequences, and the number of assembled contigs. More than 98% of the full *V. fischeri* genome was recovered in the assembly at a spike-in level of 5,000 copies, and ~30% was recovered from just 50 copies (Fig. S4). The 50 JPL SAF floor samples yielded highly variable numbers of sequences (mean, 3.18 × 10^5^; standard deviation, 4.63 × 10^6^). One air swab control, FC3, yielded 1.76 × 10^5^ sequences, while the other three air swab controls and all three water controls yielded <1,500 sequences. Consequently, the control swabs differed significantly in yield. Human sequences were identified and removed from the data set with Bowtie 2.2.9 (see Materials and Methods). Of the 50 JPL floor samples, 29 (58%) contained <1% human sequences, while 17 (34%) contained 1 to 10% human sequences. Taxonomic profiling results of samples with at least 10,000 processed sequences revealed high abundances of *Acinetobacter baumannii*, *Acinetobacter equi*, and *Acinetobacter johnsonii* in JPL SAF floor samples, while *A. lwoffii* was not detected by this method because there were no high-quality assembled genomes in the database.

### Comparing the SAF environment to two other low-biomass environments.

**FIG 4:**
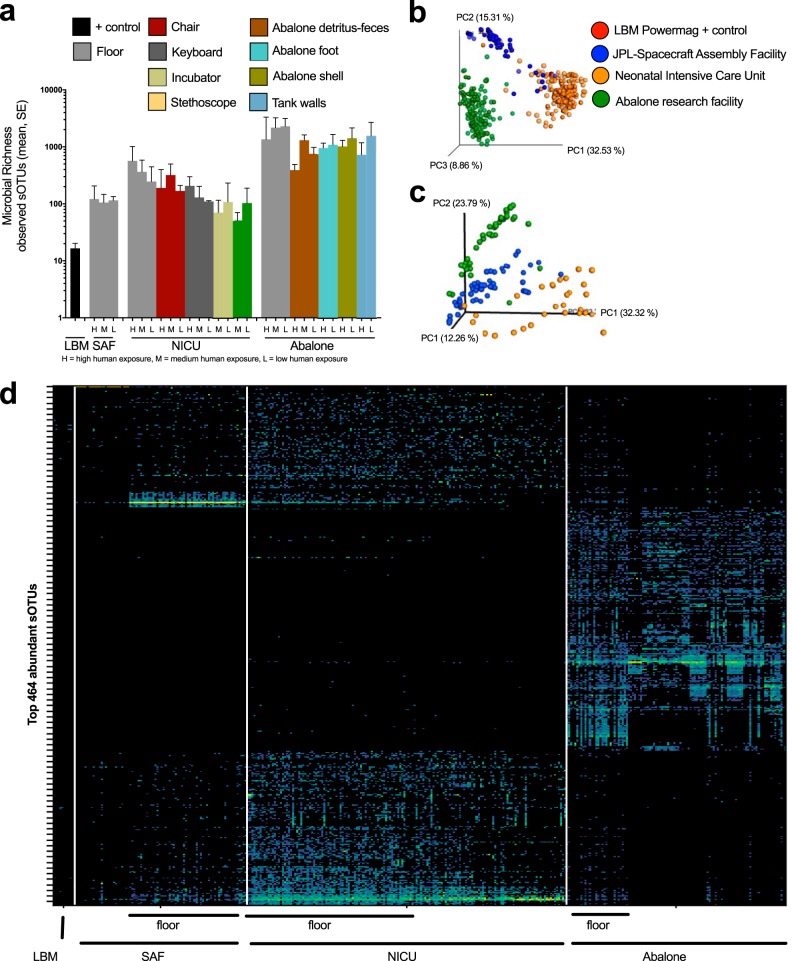
Microbial diversity was broadly compared across three unique built-environment study sites for 16S rRNA amplicon and shotgun metagenome sequencing. (a) Microbial richness of 16S rRNA amplicon data was calculated for various sample types within the built environment and organized by human exposure (H, high human exposure; M, medium human exposure; L, low human exposure). LBM, low biomass, indicates the kit testing controls from the Mobio Powermag kit. Beta diversity PCoA plots of weighted UniFrac distances of 16S rRNA amplicon (c) and Bray-Curtis distances of shotgun metagenomic samples (d) demonstrate study-specific microbial communities. The floor samples within the three built environments are solid spheres in the 16S rRNA amplicon plot (c), while other samples are transparent. (d) Heatmap depicting the top 464 of the 16,417 most abundant sOTUs across the 438 samples that passed QC. Floor samples are indicated by a line to demonstrate the similarity and differences of microbes on the floors of the three built environments.

A detailed description and interpretation of all three facilities are beyond the scope of this report, but we provide a brief comparison of the SAF data with the NICU and abalone-rearing facility data to demonstrate the utility of KatharoSeq in characterizing a range of low-biomass environments and relating them to one another (Fig. 4).

Across the three built environments, the alpha diversity measured by microbial taxon richness was highest in the abalone-rearing facility, followed by the NICU and then the SAF (Fig. 4a) (Kruskal-Wallis test statistic, 131.8; *P* < 0.0001). Within each of the three environments, human exposure was associated with microbial taxon richness, although some of the details differed among the environments. Floor samples, most exposed to direct contact with humans, consistently had the highest diversity (Fig. 4b).

In the NICU, the microbial taxon richness of keyboard, chair, and floor samples was positively associated with the human exposure of each surface. However, contrary to this general trend, the stethoscope and incubator samples had higher microbial taxon richness in low-exposure rooms. The reason for this apparent paradox is that stethoscopes and incubators are sanitized each day in occupied rooms, reducing their microbial diversity on a daily basis (Fig. 4b).

At the abalone-rearing facility, the microbial taxon richness of the abalone tank wall and floor samples was negatively associated with human exposure, contrary to the general trend. However, this effect might be due to characteristics of the influence of the individual abalone species on their surrounding microbial environments, because the abalone species are physically separated from one another (Fig. 4b). The white abalone tanks were considered to have lower human exposure, because they are physically separated from the other tanks. The total microbial taxon richness was greatest on the surface of abalone shells, followed by the foot and then the feces, which could have implications for understanding exposures of abalone to environmental microbes (Fig. 4b).

As expected and as a validation of the technique, the microbial community composition differed in each low-biomass environment, as measured by 16S rRNA analysis (Fig. 4c) and shotgun metagenome sequencing (Fig. 4d). The beta diversity distance of floor samples (solid-color spheres) from the other samples (transparent) within each built environment (by color) was smaller than the distances of floor samples from other built environments (Fig. 4c).

The NICU environment, as a whole, had greater microbial richness than the JPL SAF but less than the abalone-rearing facility. Microbial richness was highest on the floor, lowest on stethoscopes and incubators, and intermediate on chairs, keyboards, and other surfaces (Fig. 4b). Relative to the floor, the incubator was enriched in *Klebsiella* and the stethoscope was enriched in *Staphylococcus* (Fig. S4) and *Neisseriaceae*. No sOTUs were associated with acuity level or neonate occupancy when incubator, stethoscope, and floor samples were compared.

Pathogen outbreaks in hospitals are of grave concern, especially when they occur in sensitive populations such as the ICU or NICU. We were able to observe from the metagenomic data strains that were relevant to clinical increases in infection and colonization in the NICU. In the months after the study, two cases of methicillin-resistant *Staphylococcus aureus* (MRSA) invasive disease (bacteremia) were identified as an increase over baseline number of noninvasive clinical isolates of MRSA (institutional review board [IRB] number 171209X). The high-acuity F pod had six MRSA-positive cultures in 5 of the 14 rooms, while the low-acuity GH pod had only six MRSA-positive cultures across 5 of the 37 rooms (42.9% versus 23.7% incidence). In accordance with these clinical observations, we found that samples from the F pod had larger proportions of *Staphylococcus* than those from the GH pods. Since the F pod is higher acuity, it has a higher density of health care professionals, potentially contributing a higher input of *Staphylococcus*, and its rooms receive more frequent cleaning, which may maintain a harsh environment that unintentionally enriches drug-resistant bacteria. Whole-metagenome sequencing revealed that most of the staphylococcal signals were *Staphylococcus epidermidis*, although *S. aureus* was also present.

To prevent disease outbreaks and minimize patient-to-patient microbial transfer, this NICU was designed to have individual patient rooms. An outstanding question, however, is when a patient is infected, what the effect on nearby patients is. Furthermore, when a patient has an infection, does this also colonize the room itself or is it confined to the person? We observed a second strain linked to a clinical event. In the detailed survey, *Serratia marcescens*, a human pathogen associated with nosocomial infections and septicemia in the NICU (16), was highly abundant on various surfaces in room 813, including the incubator, along with various surfaces in room 811 (Fig. S3). Upon chart review, the patient in 813 developed an *S. marcescens* lung infection, which was confirmed by respiratory and eye cultures on 12 and 13 January, respectively. The patient continued to have positive cultures on 13 and 23 February 2017 after multiple administrations of antibiotics. Finding *S. marcescens* in this room in multiple sample types 48 days after the initial positive culture, while not observing the organism in two adjacent rooms, indicates the potential utility of noninvasive built-environment sampling for monitoring and discovering infectious agents in a clinical setting.

10.1128/mSystems.00218-17.3 FIG S3  Spatial microbial community maps of two common sOTUs from the NICU. Download FIG S3, TIF file, 1.9 MB. Copyright © 2018 Minich et al. 2018 Minich et al. This content is distributed under the terms of the Creative Commons Attribution 4.0 International license.

The microbial composition of the abalone-rearing facility was distinct from that of the SAF and the NICU. The abalone-rearing facility uses a flowthrough system to supply seawater to the main aquarium, while the white abalone tanks receive UV-treated and filtered seawater. Abalone hosts generally had species-specific microbiomes from the feces, the foot, and the shell (Fig. S4). When comparing microbial communities by presence or absence by using unweighted UniFrac distances, the red and white abalone body sites clustered more closely with built-environment samples taken from the respective red and white abalone tanks (Fig. S4a). Further, the pink, green, and white abalone fecal communities were more closely related than red abalone fecal communities. However, some replicates from the white abalone foot and shell mirrored that of the environment, suggesting a more transient community (Fig. S4a and b). The most abundant sOTU in the abalone aquarium microbiome was a *Vibrio* sp. (Fig. S4c). Spatially, this *Vibrio* sOTU was present across the entire facility and highly abundant in the fecal material from all of the abalone species sampled (Fig. S4c). Shotgun metagenome analysis revealed several potential *Vibrio* spp. present across the communities, with *Vibrio breoganii* followed by *Vibrio tasmaniensis* making up the majority (Fig. S4d). The other most common microbes in the facility were *Psychrilyobacter*, *Pseudoalteromonas*, *Colwelliaceae*, and *Leucothrix*, providing a microbiome markedly different from that of the other two environments sampled (Fig. 4d). The pathogen responsible for causing withering syndrome (12) was not detected in the facility or associated with the abalone hosts.

10.1128/mSystems.00218-17.4 FIG S4  Spatial microbial community map of *Vibrio* sOTU from an abalone aquarium. Download FIG S4, TIF file, 1.7 MB. Copyright © 2018 Minich et al. 2018 Minich et al. This content is distributed under the terms of the Creative Commons Attribution 4.0 International license.

## DISCUSSION

The KatharoSeq protocol integrates positive and negative controls, specific choices of high-throughput DNA extraction and bead cleanup kits, library construction and pooling strategies for amplicon sequencing or shotgun metagenomics, and bioinformatic sample exclusion protocols to achieve a high-throughput, sensitive, and specific method of probing low-biomass microbiomes. By utilizing high-throughput extraction methods, one can perform 384 DNA extractions with a single EpMotion robot paired with four KingFisher robots in 6 h, compared to processing around 72 samples per 6 h by low-throughput methods. We emphasize that positive controls both before and after DNA extraction are required, using spike-ins of different organisms so that different contamination sources can be detected and controlled. Specifically, we recommend having at least 24 total positive and 12 negative DNA extraction controls per project with at least 8 and 4 per 96-well plate. Positive controls processed before DNA extraction assess DNA extraction efficiency, provide detection limits in terms of the minimum number of cells, and reveal DNA extraction kit or sample processing contaminants. For each sequencing run, we also recommend having at least 24 to 48 PCR positive controls (12 to 24 single strain and 12 to 24 mock community) along with 8 PCR negative controls. The PCR positive controls should be a serial dilution of gDNA down to subgenome copies. Positive controls spiked before PCR amplification reveal detection limits at the PCR step, help quantify contaminants in PCR master mix reagents, and allow estimation of input DNA concentrations (Fig. 1).

We were able to easily differentiate samples from the three environments tested, and in all three cases, the shotgun metagenomic results provided a second line of evidence obtained from amplicon sequencing. For the first study, we describe the biogeography of a SAF used to construct equipment such as the Mars rovers, which has ultralow-microbial-biomass requirements. In the JPL SAF, we were able to confirm that a very plausible microbe that frequently contaminates other cleanrooms was present in this facility. *A. lwoffii* was the most common microbe found in the JPL SAF, consistent with the previous report that *Acinetobacter* was described at low levels (<5%) in the JPL SAF and at higher levels (2 to 33%) at KSC (15). *Acinetobacter* is a frequent cleanroom contaminant because it resists sterilization procedures and disinfectants and is also frequently reported as an aerosolized infectious agent in hospitals (17). In the shotgun metagenomic analysis, *A. lwoffii* did not show up in the k-mer profile because the genome of *A. lwoffii* was not in the default Kraken database, but alignments directly with the reference genome confirmed its presence. On the basis of the metagenomic approach, we also detected multiple fungal and viral groups, which is beyond the scope of standard 16S rRNA amplicon protocols. Only the careful selection of positive controls in the KatharoSeq protocol allows confidence in the identification of cleanroom microbes while controlling for kit and sample processing contaminants. By realizing the species level resolution and comparison across spatially diverse samples, we can begin to understand the origin and sources of strains in a cleanroom or hospital setting and engineer systems that eliminate these organisms.

The largest hospital microbiome study to date (7) suggests that the microbial communities of patient rooms are most influenced by the original microbes of the patients themselves. Because the neonates in the NICU do not yet have established microbiomes, we suspect that the skin-associated microbes found in the NICU are instead from health care professionals or parents. Supporting this observation, no sOTUs were associated with acuity level or neonate occupancy. Currently, methods for pathogen monitoring are restricted to patient samples that are sent for culturing only. Being able to monitor and predict pathogen occurrences by routine culture-independent noninvasive sampling of the built environment could be a useful approach for preventing outbreaks by identifying compromised or infected patients or potential hot spots of transmission. Future work should aim to establish baseline MRSA skin or nasal colonization rates of neonates and relate to infection rates. Standardized procedures need to be developed to eliminate sampling biases for sampling microbiomes paired with clinical culturing. Finding *S. marcescens*, a human pathogen associated with nosocomial infections and septicemia in the NICU (16), in a specific NICU room in multiple sample types 48 days after the initial positive culture but not in two adjacent rooms indicates the utility of KatharoSeq for biosurveillance monitoring. Similarly, aquaculture facilities often employ costly engineering controls to minimize intraunit pathogen transfer from humans, but our results suggest that most microbes in a cleanroom aquarium likely originate from the water or marine animals rather than humans. Each *Vibrio* species we observed has only been recently discovered. *V. breoganii* is a mollusk-associated, nonmotile, alginolytic marine bacterium within the *Vibrio halioticoli* clade (18, 19), and *V. tasmaniensis* was isolated from fish (19). Several *Vibrio* species have been identified as molluscan pathogens (18), and further studies should therefore be conducted to elucidate the symbiotic or pathogenic role of these species within abalone. By establishing that abalone have species-specific microbial communities with a large core of taxa shared with their environment, future studies should be aimed at understanding how perturbing the built environment can influence host microbiomes.

Taken together, our results demonstrate that the KatharoSeq protocol provides compelling microbial community analyses down to limits of detection of 50 to 500 cells in a high-throughput setting. It enables low-biomass investigations in a wide range of areas. The total processing time from when biological samples are received to when sequencing data are obtained is approximately 48 h (20). The pipeline can be easily scaled to increase throughput, because multiple steps, including plate loading, DNA extraction, PCR, and sequencing, can be automated by using robotic liquid handlers. In particular, high-throughput analysis of epidemiological cohorts of banked plasma samples and household dust samples improved the detection and control of microbial contamination in semiconductor and spacecraft fabrication settings, including planetary protection applications, and forensic analysis of degraded or minuscule samples will greatly benefit from the utilization of this high-throughput, scalable protocol.

## MATERIALS AND METHODS

### Kit comparisons.

An isolate of *B. subtilis* 3610 cultivated at 37°C overnight was sorted by flow cytometry on a Sony SH800Z FACS by using SYBR green fluorescence, isolating 5, 50, 500, or 5,000 cells in a sterile 0.22-µm-filtered phosphate-buffered saline (PBS) solution. DNA extractions from each set of sorted cells were performed in triplicate with and without carrier RNA (Thermo, Fisher catalog no. 4382878) with manual single-tube DNA extraction kits (Mo Bio PowerSoil, where the consumables were autoclaved for 3 h [21], and the Qiagen Ultra Clean kit) (Fig. 1a). A 1-µg sample of carrier RNA was added to samples after lysis. Negative controls of the PBS with and without carrier RNA along with complete blanks were DNA extracted in triplicate alongside the positive controls as well. These single-tube manual protocols were compared to the high-throughput DNA isolation protocols adopted from the Earth Microbiome Project by using the same cultures of *B. subtilis* (*n* = 9) with and without carrier RNA along with negative controls of PBS (*n* = 12) and blanks (*n* = 12) with the EpMotion 5075 liquid-handling robot (for the initial lysis steps), followed by the KingFisher Flex robot (bead-based DNA extraction). Two different bead cleanup protocols, Swiftmag beads and ClearMag beads, were tested (Fig. 1a).

All extracted DNA was amplified in triplicate for 35 PCR cycles with a 5-µl input in a 25-µl reaction mixture volume with the Earth Microbiome Project standard 16S V4 515f/806rB bar-coded primers (22, 23). These amplicons were pooled and run on 2% agarose gel and quantified with PicoGreen to access quality and relative quantity. Samples with no visible band were pooled at equal volumes of 20 µl; otherwise, samples were pooled at 50 ng per sample for a total sample volume of up to 20 µl. Sequencing was performed on the Illumina MiSeq at 2 × 150 bp by using paired-end reads. Bioinformatic processing of samples was conducted in Qiita with QIIME v1.9.1 (24) with the first read trimmed to 150 bp and then processed with deblur (25), a *de novo* sOTU picking method. For phylogenetics-based distance comparisons of the microbiomes, a tree was constructed from the sOTUs with SEPP (SATé-Enabled Phylogenetic Placement; https://github.com/smirarab/sepp/blob/master/sepp/tree.py), and then results were visualized in EMPeror (26). Sample communities were then annotated with Greengenes. The per-sample read counts generated after demultiplexing were used to compare false-negativity rates for using a gel to qualify low-biomass libraries (Fig. S2). The reference hit biom table was generated with deblur (25). The counts of all sOTUs generated from the deblur biom table were summed to determine the number of reads per sample. To determine if read counts could be used as a proxy to measure starting biomass and thus estimate sample success for other samples, read counts from positive DNA extraction controls were compared against blanks by using a nonparametric Kruskal-Wallis test with a Benjamini-Hochberg false-discovery rate (FDR) of 0.05 (Fig. 1b). The proportion of sequences aligning with *Bacillus* was measured in each sample group and also compared in the same method (Fig. 1d). Samples from the top-performing kit were then selected to compare read counts against microbial composition and fitted with an allosteric sigmoidal equation that determines the read number at which 50% of the reads align with the correct target, *Bacillus* (Fig. 1c). This was repeated again for comparing input cell counts to composition to determine the absolute limit of detection of the process (Fig. 1e). On the basis of these results, we have outlined the steps needed to perform a low-biomass microbiome analysis that includes controls at each step of the processing paired with bioinformatic analysis for sample exclusion based on read counts (Fig. 2). We applied this method to the 16S rRNA amplicon data from the three unique built environments. Sample exclusion was conservatively calculated and applied for each data set by removing samples that had less than the median read count of 5,000-cell positive controls. The success rate of 16S rRNA amplicon analysis across the three built environments was determined by the proportion of samples with read counts greater than the median read counts of the 5,000-cell positive controls. This is a conservative estimate of success, as we were able to detect positive controls down to 50 cells and thus could use the median read counts at 50 cells. Since fewer positive controls were used for the shotgun metagenome analysis, we determined success by the proportion of primary samples with read counts great than the median read counts of the negative controls. Alpha diversity (microbial richness expressed as the number of observed sOTUs) and beta diversity (unweighted and weighted UniFrac distances [27]) were calculated with Qiime v1.9.1. Beta diversity and principal-component analysis (PCoA) plots for the shotgun metagenomes were calculated by using the Bray-Curtis distance matrix (28).

### JPL SAF sample collection and processing.

Patches of surface in an ISO class 7 cleanroom at the JPL SAF in Pasadena, CA, were collected on 31 August 2016 with ultraclean water-moistened sterile swabs. Each patch was 25 cm^2^. Prior to sampling, technicians donned cleanroom garments and two sets of people were dispatched to collect samples from the JPL SAF where most of the Mars 2020 spacecraft components will be assembled. Each set of technicians consisted of two members, one of whom actively collected samples from JPL SAF surfaces while the other carefully cut the swab heads with a sterile scalpel after sample collection. One set of technicians collected samples from 50 surfaces, and a second set of technicians sampled another 50 JPL SAF surfaces. Immediately after samples were obtained, vials containing sample swabs were frozen at −80°C and sent to the University of California San Diego (UCSD) via frozen shipment. Negative controls consisted of dry swabs exposed to air in the facility for the duration of a typical sampling event, and the same molecular-grade water was used to wet the swabs.

### NICU sampling.

Patches of surface (25 cm^2^) in the NICU of the recently built (5-month-old) Jacobs Medical Center were sampled with swabs on 1 March 2017 as described in the JPL SAF facility protocol. Broad sampling of the inside of the incubator, the stethoscope surface (15 cm^2^), and the floor near the bed was done throughout all 52 patient rooms in two wings of the hospital, for a total of 156 samples. Each wing of the hospital was of either high acuity (rooms 801 to 814) or low acuity (rooms 815 to 852), where the patient-to-nurse ratio varied from 1:1 to 2:1 or 3:1, respectively. In each of the two wings, four replicate locations within the primary nursing station were sampled, including the chair seat, table, keyboard, and floor, for a total of 16 samples per nursing station or 32 samples overall. An additional 21 samples were collected in seven of the rooms for a more detailed survey of the floor, surfaces, incubator, and parent chair microbial communities. The seven rooms included two in the low-acuity wing (837 and 838) and five in the high-acuity wing (801, 802, 806, 813, and 814). Rooms 801 and 802 were both considered negative-pressure rooms and are used for quarantine purposes. Within each wing, rooms were chosen to be approximately the same distance from the nursing station while varying in occupancy, as noted by the black silhouette of a baby in the image. Within the patient room, the six surfaces swabbed included an air vent, a nurse’s chair, a nurse’s keyboard, a feeding pump or heating device, a sink, and the opening of the diaper trash can. Six surfaces of the parent’s chair included the two arm locations, seat, back, top, and behind that would not be in contact with visitors. In addition to the incubator sample taken from the inside near the head position across all 52 rooms, an additional three samples were swabbed within the incubator at the hands and feet, along with one swab from the outside that would not be in contact with a neonate. The additional floor samples were taken from each corner of the room along with the first step inside the doorway. Statistical analyses of the 16S rRNA amplicon and shotgun metagenome data were performed as described in Materials and Methods. The 388 samples were processed by the high-throughput methods and then 16S rRNA amplicon sequenced. Further, 337 samples processed through the high-throughput KatharoSeq protocol were processed for shotgun metagenome sequencing.

### Southwest Fisheries Science Center abalone research aquarium sampling.

Abalone-rearing facility samples were taken on 14 and 24 February 2017 at a total of 159 sampling points, including 68 abalone-associated and 91 facility- or tank-associated samples (Fig. S4). Within the facility, seawater is filtered to remove larger (50- to 100-µm) particles, UV treated, and then ozonated to remove and destroy microbes from the water before deployment to animal tanks. Ten individual white abalone were weighed, measured for length, sexed, and then swabbed on the foot and shell, and four fecal detritus pellets were removed from the tank, for a total of 24 white abalone samples. Eight red abalone were swabbed on the foot and shell, and eight fecal pellets were collected from the bottom of the tank, for another 24 samples. Five green abalone fecal pellets were collected, and five fecal pellets were collected from three different pink abalone tanks. Each of the three pink abalone tanks represented animals that had undergone oxytetracycline antibiotic treatments at various times in the past (1 day, 90 days, or 3 years). Forty-one facility samples included 18 floor samples taken at two time points along with a door handle, two skimmer locations, and two table surfaces. Twenty-seven samples were taken from the white abalone tank, including five bottom, four turf liner, four wall, five PVC pipe, one air hose, four water, and four kelp food samples. Twenty-three samples were swabbed from the red abalone tank, including four tank lip (not touching water), two PVC pipe, four air stone, five water, four wall, and four kelp samples. All samples were processed through the KatharoSeq protocol, with equal volumes pooled and processed for 16S rRNA amplicon and shotgun metagenome sequencing. The work involving white abalone at the Southwest Fisheries Science Center was permitted under ESA permit no. 14344-2R issued to J. Hyde. Sample swabbing occurred concurrent with regular husbandry and health assessment monitoring that together was deemed a *de minimus* activity and did not require IACUC approval.

### DNA extraction.

On the basis of the protocol testing results, DNA extraction was performed in accordance with the KatharoSeq protocol (Mo Bio PowerMag kit with ClearMag beads) and quantified down to 1 pg/µl with the Qubit (Thermo, Fisher). DNA-positive controls consisted of *B. subtilis* flow sorted into 5, 50, 500, or 5,000 cells (*n* = 3 each). Blanks with no cells were also included.

### Amplicon library construction.

16S rRNA amplicon PCR amplification was performed as described in the KatharoSeq protocol in Fig. 2, with all amplicons pooled at equal volumes of 20 µl and sequenced on the Illumina MiSeq at 2 × 150-bp paired-end reads. Sample volumes were equally pooled regardless of the presence of an amplicon band to reduce false negatives (Fig. S2) and as a comparative measure of the relative starting DNA biomass. PCR positive controls consisted of *V. fischeri* at 0.1 to 500,000 genome copies (estimated by Qubit DNA concentration). Internal transcribed spacer PCR amplification with bar-coded primers ITS1f/ITS2 was also performed, but all samples failed to produce any detectable library and thus were not sequenced.

### Amplicon data analyses.

*De novo* sOTU picking was performed in Qiita (qiita.microbio.me) and QIIME as described above. Microbial richness was measured by determining the number of observed sOTUs per sample. The mean compositions of highly abundant OTUs from the QIIME analysis of the JPL SAF floor samples and across the various positive and negative controls are summarized in Fig. S3.

Finer-grained *de novo* sequence analysis was performed by deblur (25) and visualized in Calour (http://github.com/amnona/calour), providing single-nucleotide polymorphism resolution (Fig. S3). Microbial diversity was calculated by determining the total number of observed sOTUs per sample. Differential abundances of JPL SAF floor microbes were compared to JPL positive controls by using a permutation-based group mean comparison with the FDR controlled to 0.05 by using the Benjamini-Hochberg procedure (29) within Calour (Fig. S3). Spatial mapping was performed with ili, a toolbox for molecular mapping in two and three dimensions (30) (Fig. 3d). To evaluate a deeper taxonomic resolution on the JPL SAF samples, we processed the JPL plates through the Kapa Hyper Plus library prep protocol. Only one of the two plates had enough DNA after library preparation to load onto the HiSeq Rapid Run 2 × 250-bp run.

### Shotgun metagenomic data analyses.

The sequencing data were processed through Trimmomatic 0.36 (31) to trim off low-quality reads and Bowtie 2.2.9 (32) against human reference genome assembly GRCh38 to remove potential human contamination. Taxonomic profiling was conducted with Kraken 0.10.5 (33) against a database that contains all of the complete genomes in the NCBI RefSeq database under the categories bacteria, archaea, viral, protozoa, fungi, and human as of November 2016. Relative abundances of taxa were computed by dividing the sequences assigned to this taxon against the total number of processed sequences. Selected *V. fischeri* gDNA-spiked samples were subjected to *de novo* assembly with SPAdes 3.9 (34). The resulting contigs were qualified with QUAST 4.3 (35) and further scaffolded with Ragout 1.2 (36) against the *V. fischeri* ES114 reference genome (RefSeq accession no. GCF_000011805.1). The resulting draft genomes were aligned with the reference genome by using Brig 0.95 (37). Default parameter settings were used for these programs unless otherwise stated.

### Data availability.

Data from all of the experiments described here have been made publicly available in Qiita (Qiita study ID 10934, prep 3975, https://qiita.ucsd.edu/study/description/10934) and ENA (RefSeq accession no. EBI ERP105802).

10.1128/mSystems.00218-17.5 FIG S5  Alignment of draft *V. fischeri* genome assembled from spiked controls with the reference genome. Download FIG S5, TIF file, 1.3 MB. Copyright © 2018 Minich et al. 2018 Minich et al. This content is distributed under the terms of the Creative Commons Attribution 4.0 International license.
