# Supplementary figures and images for: KatharoSeq Enables High-Throughput Microbiome Analysis from Low-Biomass Samples
Source: mSystems. 2018 Mar 13;3(3):e00218-17. doi: 10.1128/mSystems.00218-17 (PMC5864415; doi:10.1128/mSystems.00218-17)

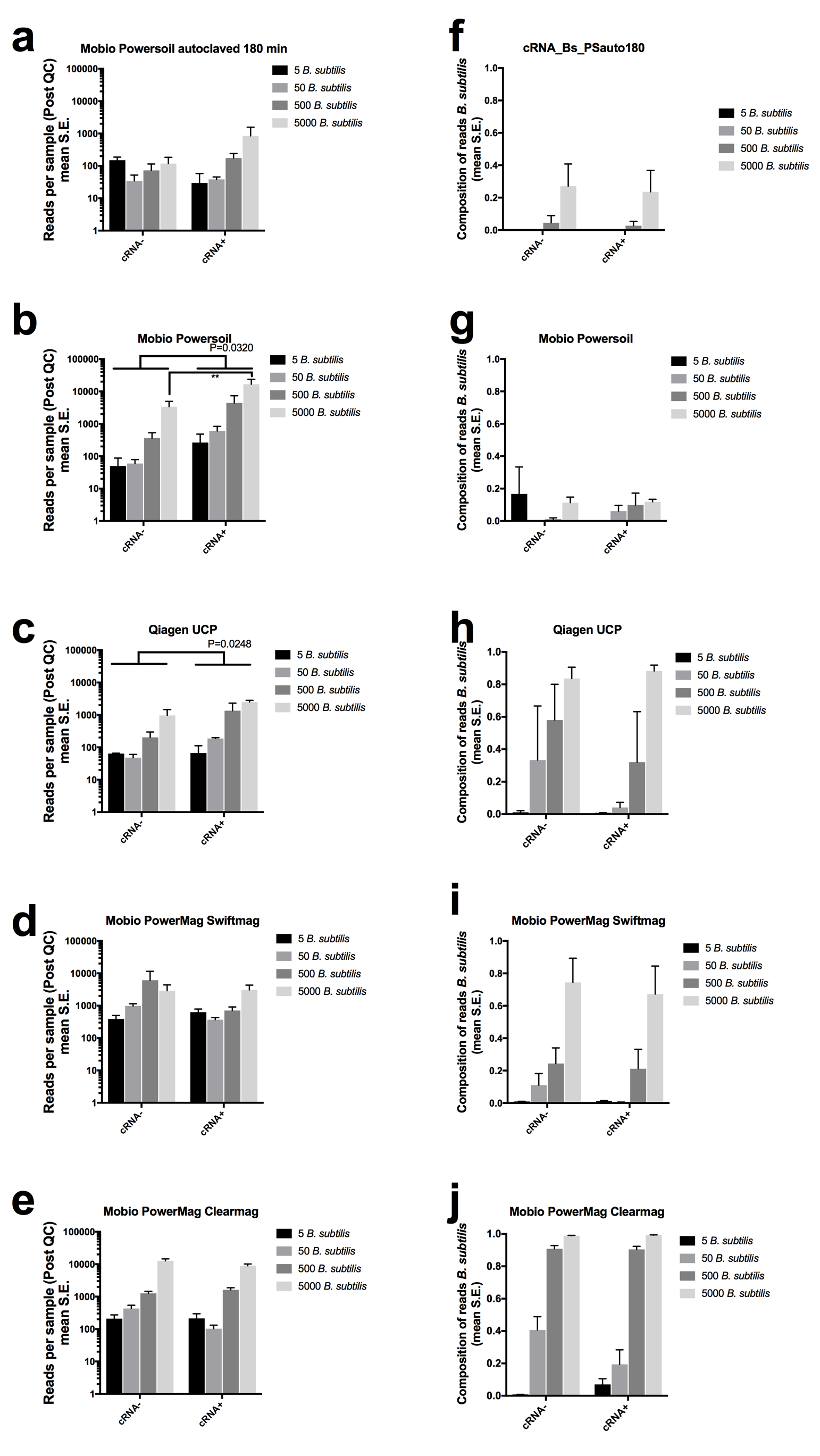

Supplement: FIG S1 [file sys001182194sf1.tif]

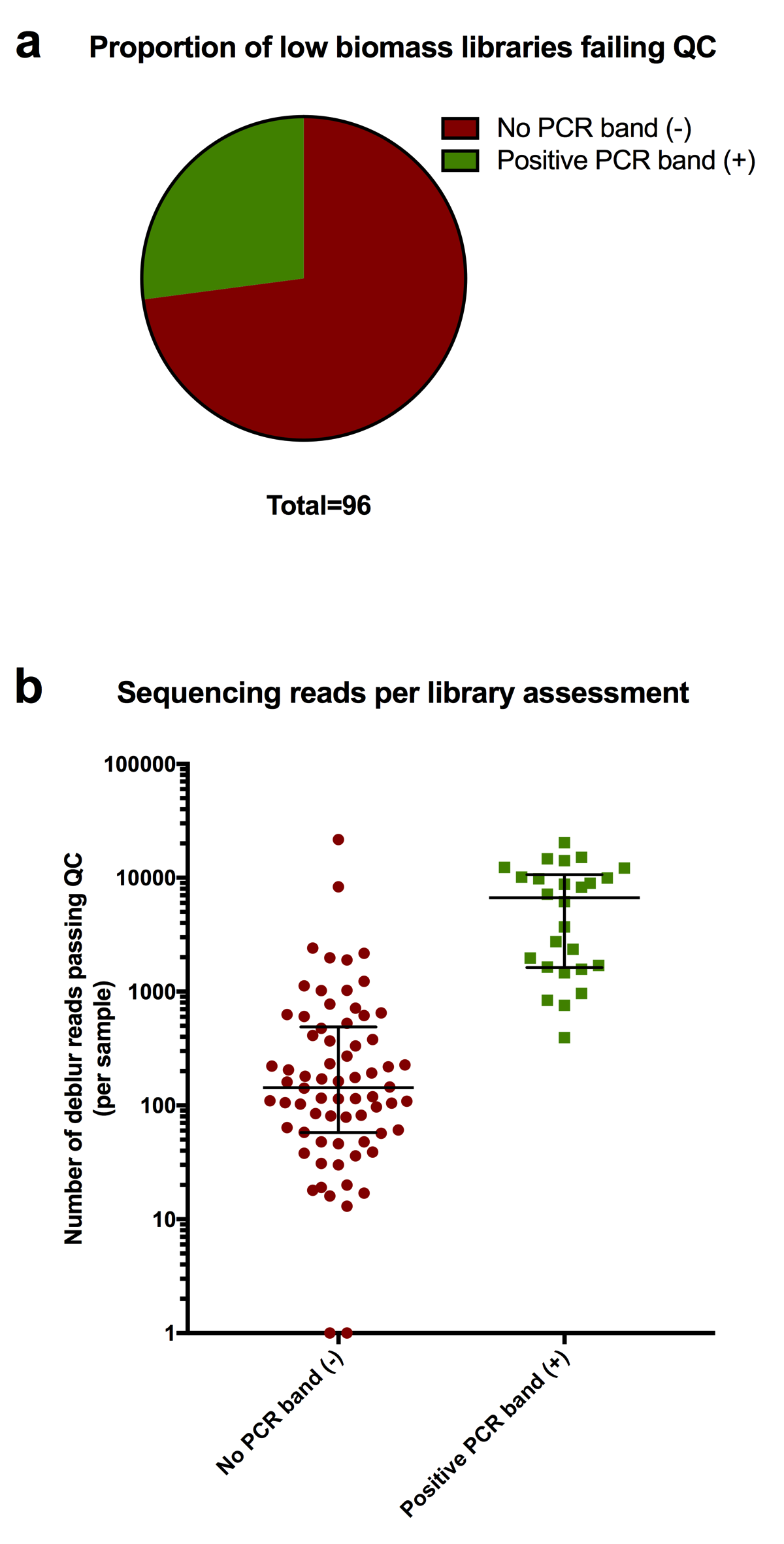

Supplement: FIG S2 [file sys001182194sf2.tif]

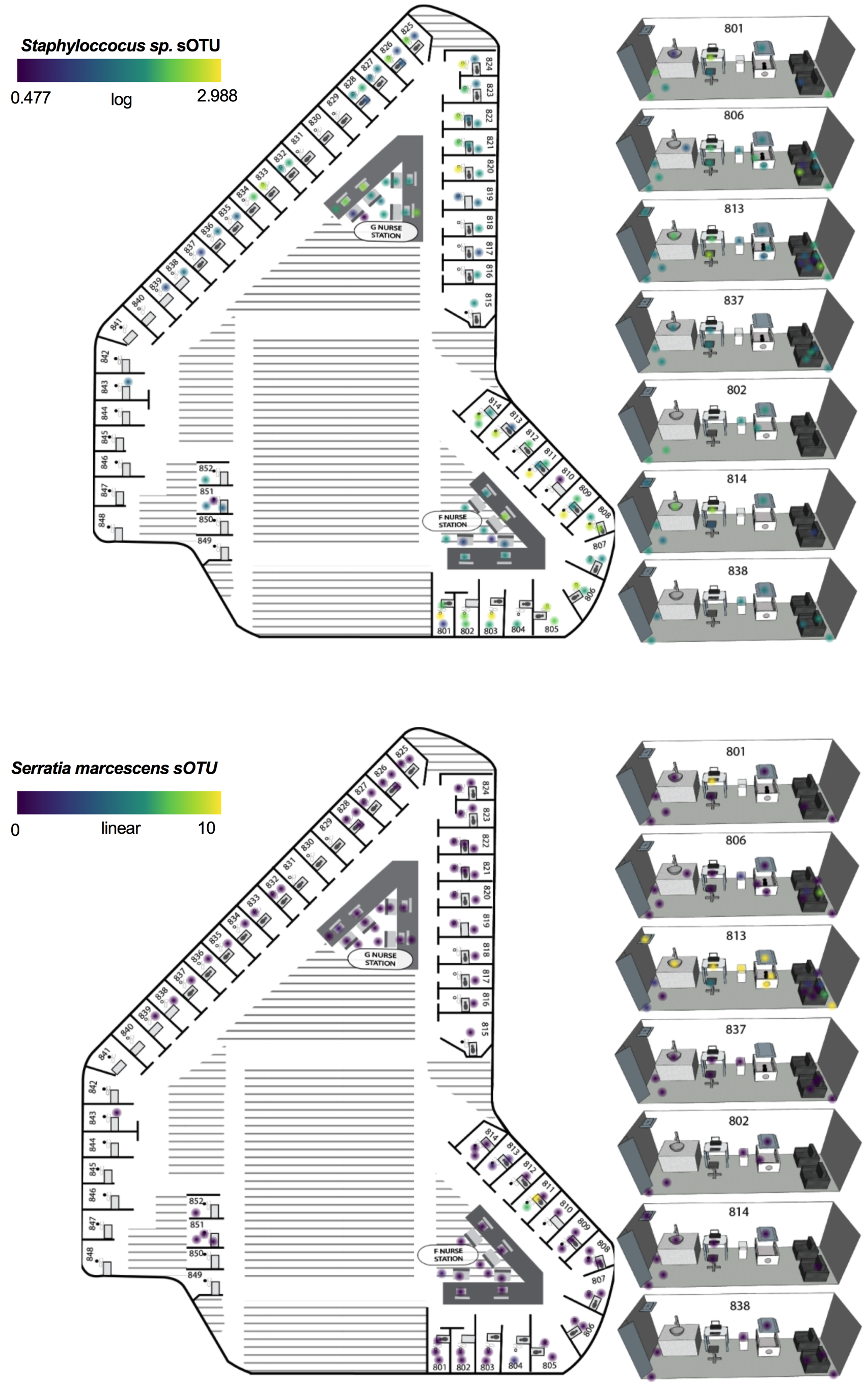

Supplement: FIG S3 [file sys001182194sf3.tif]

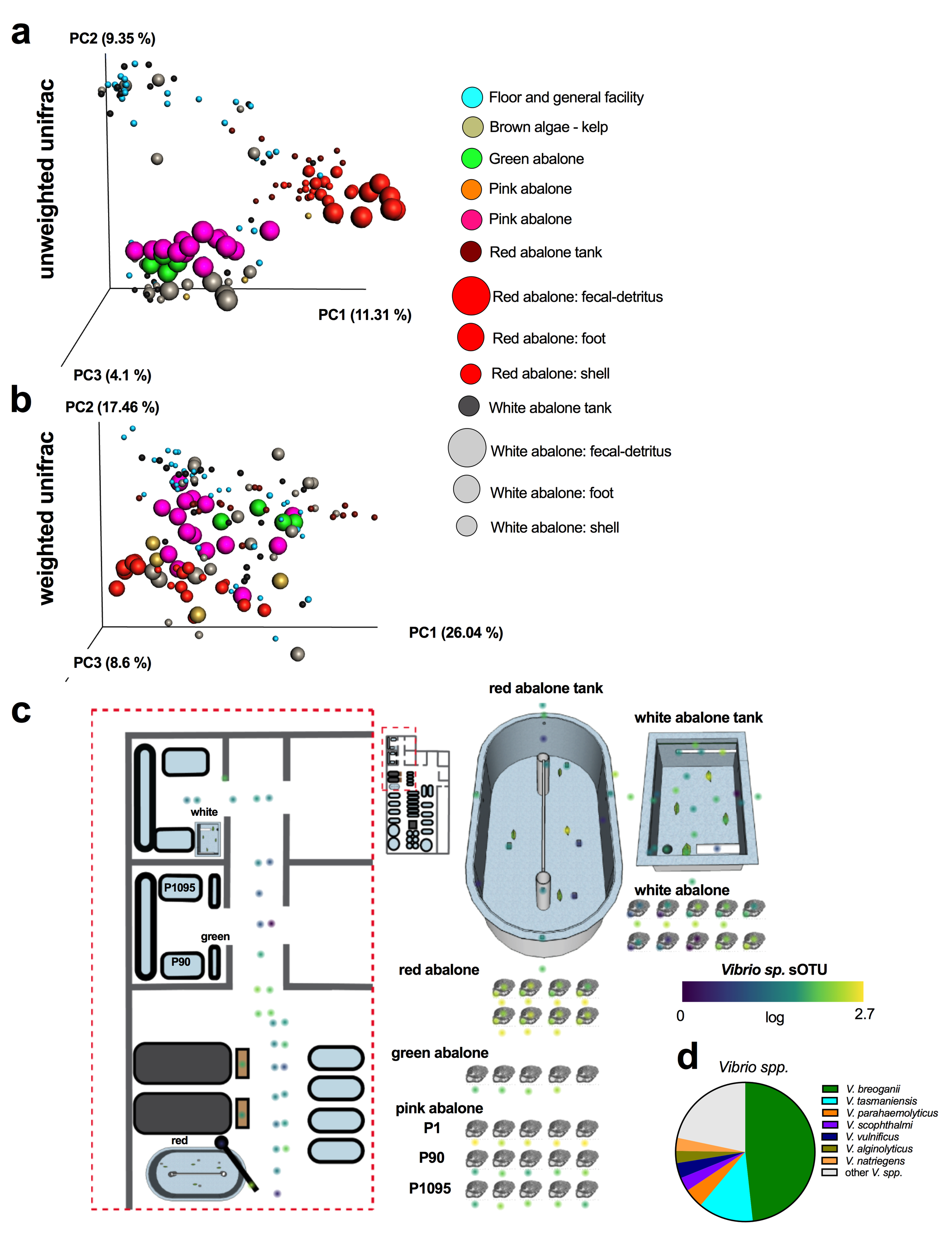

Supplement: FIG S4 [file sys001182194sf4.tif]

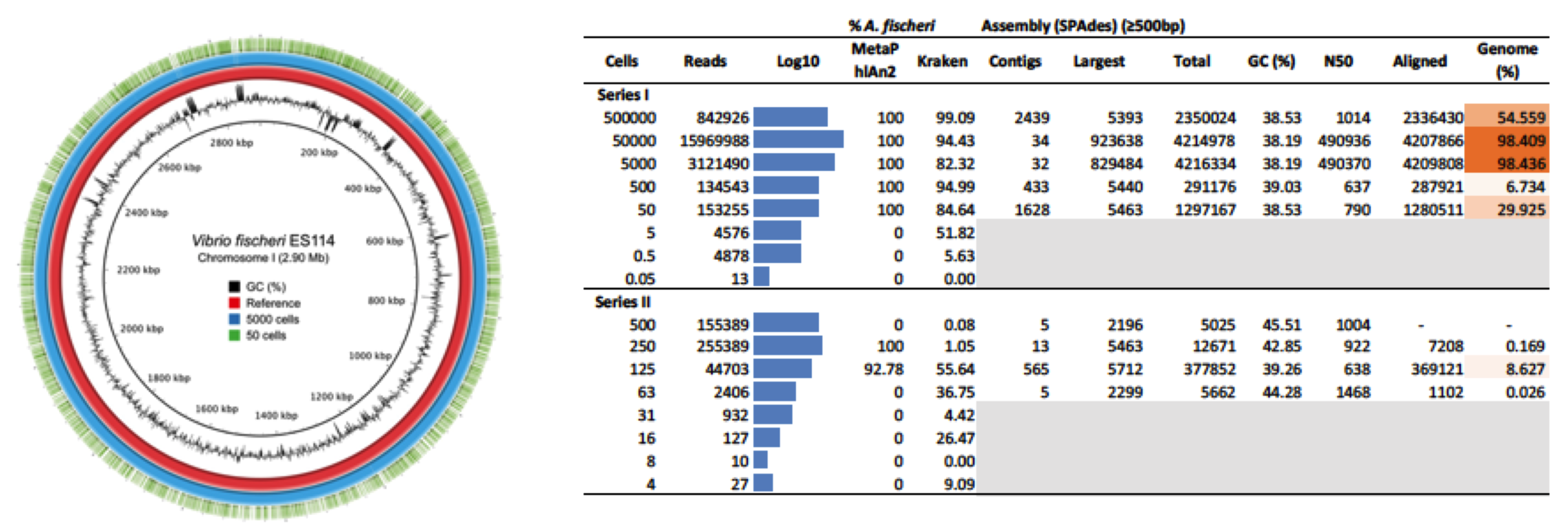

Supplement: FIG S5 [file sys001182194sf5.tif]
